# Steaming Maintains Fatty Acids, Antioxidants, and Proximate Content in Snack Bar Products from Cocoa Beans

**DOI:** 10.1155/2024/1406858

**Published:** 2024-03-12

**Authors:** Kasma Iswari, Atman, Leni Marlina, Riska, Sri Satya Antarlina, Ratna Wylis Arief, Waryat, Rosniyati Suwarda, Noveria Sjafrina, Alvi Yani, Sunarmani, Gabriel Herald Joseph, Meivie Lintang, Payung Layuk, Abdullah Bin Arif

**Affiliations:** ^1^Research Center for Agroindustry, National Research and Innovation Agency, Bogor, Indonesia; ^2^Research Center for Horticulture, National Research and Innovation Agency, Bogor, Indonesia; ^3^Research Center for Food Technology and Processing, National Research and Innovation Agency, Yogyakarta, Indonesia

## Abstract

Chocolate products on the market are generally in the form of chocolate bars as snacks made from cocoa powder. Fat and powder are separated first through a pressing process to obtain the cocoa powder. Cocoa powder loses most of its fat content during processing. Therefore, the study aimed to determine the effect of steaming time on the cocoa bean content of fatty acids, free fatty acids, proximate levels, and antioxidant activity of snack bar products made from steamed cocoa beans. Seven steaming time intervals for cocoa beans were studied. The results showed that a longer steaming time affects the fatty acids, saturated fatty acids, antioxidants, and proximate in cocoa beans. Steaming time treatment at 45 minutes increased oleic acid, palmitic acid, and antioxidant activity. In addition, reducing free fatty acids represents a quality improvement that meets international Codex Alimentarius standards, offering a competitive advantage in the market. The food industry can adopt this steaming technique to develop snack bars and new products that are healthier and more sustainable by using steaming as an effective processing method in maintaining and increasing the nutritional value of products.

## 1. Introduction

Chocolate products made from cocoa beans (*Theobroma cacao*) are popular worldwide because of their delicious taste and distinctive aroma [1]. Cocoa beans contain 35–50% fat, which consists of fatty acids, 15% starch, 15% protein, 1–4% theobromine, and 0.07–0.36% caffeine, and 0.19–0.30% theobromine, and the epidermis contains about 0.19–2.98% alkaloid compounds, 0.05–0.36% caffeine compounds, tryptophan, and essential amino acids, which are serotonin precursors, functioning as neurotransmitters involved in regulating mood [2–6]. Therefore, cocoa-based foods are rich in compounds that are beneficial to health and are considered functional foods [7]. Functional foods can function as anti-inflammatory, antioxidant, antimicrobial, analgesic, and vasodilator properties and also contain Ca, Fe, Mg, Mn, K, and Zn as sources of essential elements in the body's metabolism [8, 9].

Chocolate products on the market are generally in the form of chocolate bars as snacks made from cocoa powder [10]. To obtain the cocoa powder, fat and powder are separated first through a pressing process [11]. In this process, beneficial compounds are lost in cocoa powder as an essential product ingredient because they are released during compression along with fat. However, cocoa powder loses most of its fat content during processing [12]. During the transformation of fresh cocoa beans to cocoa powder, the concentration of bioactive compounds can be affected by various biological and processing conditions, where it decreases during transformation. Cocoa butter is rich in antioxidants that can fight free radical damage to the skin [5]. Free radicals cause premature ageing, dull skin, and dark spots on the skin and cause other degenerative diseases [8, 13].

Consumer demand for snack products that benefit health is increasing [7, 14]. Snacks are an essential part of the human diet, providing a feeling of satiety and supplying the necessary nutrients [15]. In addition, snacks also function as healthy food containing amino acids, vitamins and minerals, fatty acids, and sufficient levels of proximate and low levels of free fatty acids, where these compounds are needed by the human body [15]. The product's full free fatty acid content is only 1.7% [16] because free fatty acids cause rancidity in products [5].

One of the snack products is a snack bar. Sarkkinen et al. [17] stated that snack bars are snacks or blocks made from cereals, nuts, or other ingredients. The snack bar is a type of processed food with solid characteristics and is made from a combination of several food ingredients, combined into one with the help of a binder. Binders can be in the form of syrups, caramels, chocolates, and others. The advantages of snack bars include extended shelf life, high-calorie content, and not easily damaged in distribution [15].

Technology to increase the availability of cocoa products in the community with better health benefits is urgently needed, including the steaming of cocoa beans. Bai et al. [18] have proven that the steaming treatment produces better-quality raw materials and can maintain quality, especially by suppressing free fatty acids during storage in oat bran. In addition, Jia et al. [19] have also proven that the effect of steaming on wheat as a raw material for wheat noodles can increase the fatty acid content of wheat noodles during storage. Therefore, the steaming technique has great potential for preserving the beneficial compounds in cocoa beans. However, the study of steaming in cocoa beans still needs to be improved. Previous research generally used superheated steam roasting, a tool for roasting cocoa beans by blowing hot steam [20]. Superheated steam roasting technology is a modern technology that uses modern tools; therefore, it cannot be applied at the farmer level or small and medium enterprises (SMEs).

Therefore, the study aimed to determine the effect of steaming time on the cocoa bean content of fatty acids, free fatty acids, proximate levels, and antioxidant activity of snack bar products made from steamed cocoa beans. The results of this study can be utilized by the food industry, especially the small and medium industry (SME) level, so that the number of food industries can be increased. An increase in the number of food industries using cocoa as raw material will increase the demand for cocoa beans and impact the selling price of cocoa, ultimately increasing the income of cocoa farmers.

## 2. Materials and Methods

### 2.1. Plant Material

Dried fermented cocoa beans BL-5 were obtained from the Belubus Agricultural Technology Park, Limapuluh Kota Regency, West Sumatra Province, Indonesia. BL-50 is a local variety resulting from the purification of cocoa farmers, with a moisture content of 7.5%. Cocoa was harvested during the peak harvest season between August and October. Fermentation of cocoa beans was carried out by spontaneous fermentation. The fermentation process was carried out for five days. The cocoa beans were stirred every two days to smooth the fermentation process. Then, the fermented cocoa beans were air-dried. The cocoa beans were sorted by separating the defective and slaty beans. 21 kg of sorted cocoa beans was used as raw material for snack bar products.

### 2.2. Cocoa Nib Bar Production Process

**Figure 1:**
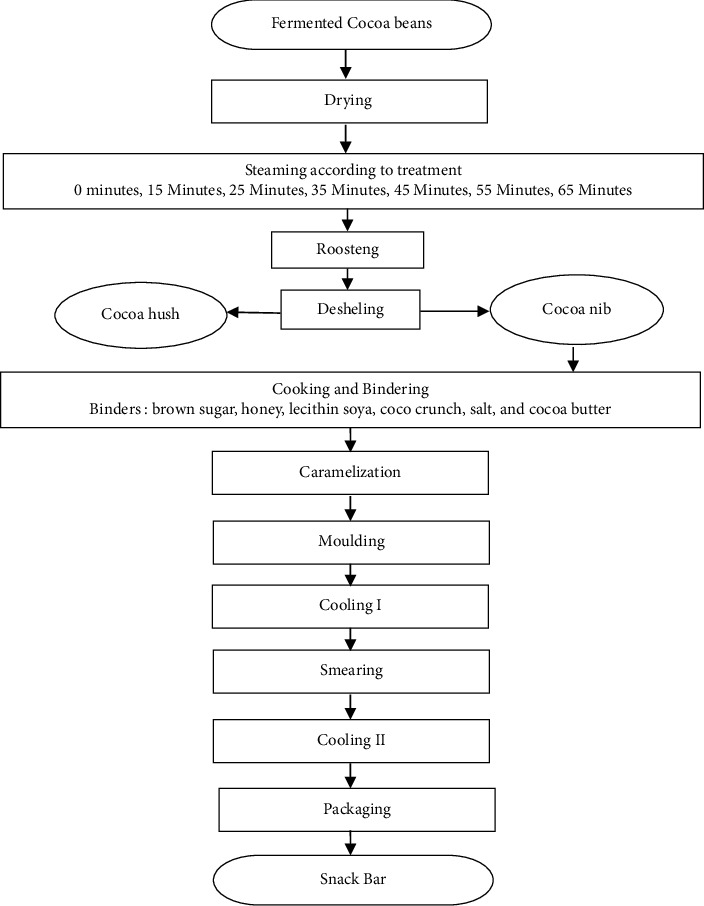
Flowchart for the manufacture of snack bars.

Three kilograms of cocoa beans was weighed and steamed for each test sample. There were seven intervals of steaming cocoa beans tested: 0, 15, 25, 35, 45, 55, and 65 minutes which were repeated three times. Steaming was performed using a stainless steel steamer with a size of 40 cm at boiling temperature (100°C). Then, the cocoa beans were air-dried, and the cocoa beans were roasted using a rotary flat cylinder-type roaster with a capacity of 5 kg. The cocoa beans were broken, and the husk was removed using a Deshler to obtain the cocoa nib in the form of granules. Furthermore, the cocoa nib added the ingredients as a binder: brown sugar, granulated sugar, honey, lecithin soya, coco crunch, salt, and cocoa butter. Then, it was cooked and molded, as shown in the flowchart process in Figure 1. The variables included proximate content (moisture, ash, fat, protein, and carbohydrates), free fatty acids, essential fatty acids (myristic, palmitic, stearate, oleic, linoleate, and linolenic), and antioxidant activity (IC_50_).

### 2.3. Fatty Acid

The determination methods of fatty acids refer to the reported study with modification [21]. Fatty acid analysis was performed using gas chromatography (Hitachi Type 263-50) according to IUPAC Procedure 1987-2401. The results were expressed as percentages.

### 2.4. Antioxidant Activity

Analysis of antioxidant activity as a free radical scavenger 1,1-diphenyl-2-picrylhydrazyl (DPPH) referred to the method described by García-Alamilla et al. [22]. The extract solution was made at a concentration of 5–40 ml/l. The extract solution was taken as much as 1 ml, and then, we added 2 ml of 0.1 mM DPPH solution (DPPH in methanol). The mixture was vortexed and calibrated at a wavelength of 517 nm with a spectrophotometer, then allowed to stand for 30 minutes at room temperature in a dark room, and calibrated at 517 nm. Aquadest 80% was used as a control, and BHT and ascorbic acid were used to compare. The difference in absorbance of the sample and control indicates the effectiveness of DPPH radical scavenging. The formula calculated the percentage of free radical scavenging activities:

(1) $$\begin{array}{c} \text{DPPH }\left(\%\right)=\frac{\text{Control absorbance}-\text{sample absorbance}}{\text{Control absorbance}}\times 100\%. \end{array}$$

### 2.5. Determination of Proximate Content

#### 2.5.1. Moisture Content (Gravimetric Method)

The mashed sample was weighed as much as 5 g and dried in an oven at 105°C for 5 hours [23]. The sample was cooled in a desiccator and weighed. Then, it was heated in the oven for 3 minutes, cooled in a desiccator, and weighed. This treatment was repeated until a constant weight was reached (weighing difference of 0.2 mg). Weight reduction is the amount of water in the material. The calculation of moisture content based on dry weight is as follows:

(2) $$\begin{array}{c} \text{moisture content}\left(\%\right)=\left(\frac{i\text{nitial weight}-\text{final weight}}{i\text{nitial weight}}\right)\times 100\%. \end{array}$$

#### 2.5.2. Ash (Thermogravimetry Method)

An ashing dish (porcelain crucible) was prepared and then heated in an oven at 105°C [24], cooled in a desiccator, and weighed. A 5 g sample was inserted in an ashing dish, put into an ashing furnace at a temperature of 600°C, and burned until gray ash was obtained or until the weight was constant. The sample was cooled in a desiccator and then weighed. The calculation of ash content is as follows:

(3) $$\begin{array}{c} \text{ash}\left(\%\right)=\frac{\text{ash }w\text{eight}}{\text{sample weight}}\times 100\%. \end{array}$$

#### 2.5.3. Fat (Soxhlet Extraction Method)

We accurately weighed 3 g of mashed material, wrapped it in paper tissue, tied it with thread, and put it into a Soxhlet extraction tube in the lead [24]. Cooling water flows through the condenser. The extraction tube was mounted on a Soxhlet distillation apparatus with 35 ml of petrothelium ether as a solvent for 4 hours. The fat extract in the extraction tube containing the petrothelium ether was dried in an oven at 105°C to obtain the constant weight. The residual weight is expressed as the fat weight.

#### 2.5.4. Protein (Kjeldahl Method)

We accurately weighed the homogeneous cocoa sample (1.5 g) [24], placed it in a 500 ml Kjeldahl flask, and added a catalyst (half a Kjeldahl tablet) and 15 ml H_2_SO_4_. Kjeldahl flasks were installed in the destruction apparatus rack and destructed for 1 hour until the solution turned clear and cooled for approximately 1 hour. We added 25 ml of distilled water and three drops of pp indicator into the Kjeldahl flask through the wall. The Kjeldahl flask was transferred to a distillation unit, and 20 ml of 45% NaOH was added. The distillation results were accommodated in an Erlenmeyer flask containing 20 ml of 3% boric acid solution and three drops of methyl red indicator. Blanks were made with the treatment as above without the addition of samples. The distillate obtained was titrated with HCl (0.058 N) until the color changed to pink.

#### 2.5.5. Carbohydrate Content

Carbohydrate content calculated using the by-difference method is a reduction in of 100 for moisture, protein, fat, and ash content [24].

### 2.6. Data Analysis

Data were analyzed with a one-way analysis of variance. The significant differences among the treatment means were determined by the Duncan multiple range test (DMRT) at a probability level of 5%. Statistical analyses of data were performed using SAS Portable Version 9.1.3. The data were reported as the mean of three replications.

## 3. Result and Discussion

### 3.1. Result

#### 3.1.1. Fatty Acid

There are six main fatty acids in snack bars from cocoa beans: oleic, palmitic, stearic, linoleic, linolenic, and myristic acids (Table 1). Oleic acid is a fatty acid with the highest percentage content compared to other fatty acids. The steaming time treatment significantly impacted cocoa beans' snack bar fatty acid profiles. The fatty acid content increases with increasing steaming time and decreases after steaming for more than 45 minutes. The 45-minute steaming treatment resulted in the highest oleic, palmitic, stearic, linoleic, and linolenic acid content compared to the other steaming treatments, except for myristic acid. The distinction of fatty acid content in the 45-minute steaming treatment compared to the control were 7.32, 13.94, 1.83, 1.52, 0.08, and 0.03%, for oleic, palmitic, stearic, linoleic, linolenic, and myristic acids, respectively. In addition, the steaming time treatment resulted in a higher fatty acid content than the control.

#### 3.1.2. Free Fatty Acids and DPPH Radical-Scavenging Activity

The steaming time treatment of cocoa beans significantly affected snack bars' free fatty acids and antioxidant activity (Table 2). There was a decrease in free fatty acids and DPPH IC50 when cacao beans were treated with a steaming time of up to 45 minutes, but there was no significant decrease after 45 minutes of steaming treatment. Steaming cocoa beans can reduce snack bar products' free fatty acid content. Free fatty acids of snack bar products ranged from 0.015 to 1.090%. In addition, the steaming time treatment of cocoa beans can increase antioxidant activity, as evidenced by the decreasing IC_50_ value. This study found that steaming for 45 minutes could reduce the DPPH IC_50_ value by 68.60%, from 43.99 to 13.81 ppm.

#### 3.1.3. Proximate

The steaming time increased the moisture and fat of snack bars; on the other hand, it decreased ash, carbohydrate, and protein (Table 3). In addition, the moisture content in the control was 1.15%, which gradually increased when the steaming time was also increased. In addition, it also affected the carbohydrate content. The highest carbohydrate content obtained without steaming (control) was 42.52%, and the lowest was obtained when the beans were steamed for 65 minutes, 34.99%. The highest ash content was obtained without steaming, which was 3.15%. The ash content of cocoa beans without steaming differed significantly with all treatments. Steaming the cocoa beans for 15 minutes did not differ significantly from the treatment for 25–55 minutes. Still, it was significantly different from the treatment of steaming for 65 minutes. The longer the cocoa beans are steamed, the ash content of the product decreases. The fat content of snack bars in the 45-minute steaming time treatment tended to increase, 7.78% higher than the control. Steaming cocoa beans significantly affects the protein content of snack bars. The highest protein content was obtained in the nonsteaming treatment with a protein content of 12.61%, significantly different from all steaming treatments for 15–65 minutes. By steaming the cocoa beans, the protein content decreased.

### 3.2. Discussion

Fatty acids are one of the main components of oil. Saturated and unsaturated fatty acids play an essential role in the human body. The dominant fatty acids in snack bar products are palmitic acid and oleic acid. ElKhori et al. [25], Katz et al. [1], and Oliva-Cruz et al. [4] reported that the dominant fatty acids in cocoa nibs and their products were oleic acids (C18 : 1), stearic acid (C18 : 0), and palmitic acid (C16 : 1). However, in this study, the stearic acid content produced was lower (Table 1). In addition, Żyżelewicz et al. [26] reported that the three highest types of fatty acids were palmitic 26.4%, stearate 35.81%, and oleic 33.74%. Geographical differences and analytical methods can cause these differences [4], also depending on the growing conditions of the cocoa plants, which give different physical properties [3, 27]. Increased fatty acids are caused by steaming, which can deactivate peroxidases (PODs) and lipases, which produce lower peroxide values, thereby increasing the content of unsaturated fatty acids and improving the sensory value of the product [18]. As the interval of steaming cocoa beans increases, the level of fatty acids also increases. However, the steaming time of 65 minutes decreased fatty acid. This is because the longer the steaming time, the dry matter's weight decreases, which decreases the specific gravity of the material. Specific gravity is the total nutrient component except for water [27].

One of the primary fatty acids in cocoa beans is oleic acid (59–67%). This oleic acid belongs to the monounsaturated fatty acid (MUFA) group and has 18 carbon atoms with one double bond. Asmaa et al. [28] reported that oleic acid functions to regulate human physiological functions to prevent disease disorders; an experimental study conducted on rats showed that a high oleic acid diet derived from olive oil could increase levels of omega-three polyunsaturated fatty acids (PUFAs), and this subsequently positively correlated with reduced risk of cardiovascular disease, increased HDL cholesterol levels, and decreased triglyceride levels and body weight. Oleic acid in the steaming treatment was higher than that without the steaming treatment. The increase in oleic acid content can be caused by the release of fatty acids from cells damaged by high-temperature treatment [29]. In addition, 100–110°C temperatures will induce hydrolysis of triacylglycerols to produce free fatty acids [30]. Therefore, steaming treatment is recommended to increase the oleic acid content in cocoa beans, which is beneficial for the health of the human body.

In addition, palmitic and stearic acid content ranked second and third in total fatty acid cocoa beans, dramatically increasing by the steaming treatment (Table 1). As a saturated fatty acid (SFA), although its content increases after processing, it decreases after steaming for more than 45 minutes. Fatty acids in cocoa beans increased with higher treatment and more extended time. This is probably due to more damage to the cocoa bean cells due to the long steaming time. Therefore, the 45-minute steaming treatment increased the fatty acid content in the cocoa beans.

Free fatty acids indicate damage to the quality of food products because they can change the nutritional value of food and make food less attractive and unpleasant. In this study, free fatty acids of snack bar products ranged from 0.015 to 1.090%, which is much lower than the research results [31], around 1.81–1.92%. The longer the steaming time of cocoa beans increases, the free fatty acid product of snack bars decreases. This is because steaming will provide hot steam, which can provide an oxygen-free environment conducive to inhibiting lipid oxidation; therefore, it can suppress the formation of free fatty acids [19]. During storage, Bai et al. [18] reported that steaming decreased wheat bran-free fatty acids. It is proven from their research results that steaming treatment can reduce levels of free fatty acids. The free fatty acid content was 70, 75, and 80 KOH/100 g for steaming, microwaving, and without heating treatment, respectively. In addition, the free fatty acid content at eight weeks of storage was 75, 95, and 110 KOH/100 g for steaming, microwaving, and without heating treatment, respectively. It shows that the increase in FFA in the steaming treatment is only 7% and the lowest compared to other treatments. Good cocoa butter contains about 98% triglycerides, less 1.75% free fatty acids, 0.3–0.5% diglycerides, 0.1% monoglycerides, 0.2% sterols, 0.05–0.13% phospholipids, and small amounts of tocopherols [16, 19]. The Codex Alimentarius Commission [32] stipulated that the maximum free fatty acid content tolerance in cocoa beans and their products is 1.75%. Afoakwa et al. [16] stated that the high content of free fatty acids in cocoa beans or processed products will cause the rancidity process to occur more quickly, reducing sensory and economic value. Therefore, the amount of free fatty acids is used to measure the rancidity of the nibs and cocoa products. Free fatty acid levels are fats that damage quality parameters due to the hydrolysis process, where the triacylglycerol fatty acids produced from short chains produce a rancid aroma and taste [33]. Free fatty acids in cocoa butter must be avoided because they indicate quality damage. In this study, it can be recommended that steaming cocoa beans is sufficient for up to 45 minutes because it can reduce the free fatty acid content of snack bar products from 1.090% before steaming to 0.020% (89%) and is not significantly different from the 55 and 65 minutes steaming time.

Besides free fatty acid parameters, antioxidant activity is also an important parameter that influences the quality of cacao products. Antioxidant activity can be assessed from IC_50_, namely, the concentration of the sample solution needed to counteract 50% of the DPPH free radical (1,1-diphenyl-2-pikrilhidrazil), where the lower IC_50_ value indicates higher antioxidant activity [34]. This means that its effectiveness as a free radical scavenger occurs at a lower IC_50_ value [35]. The 45-minute steaming treatment was the best in this study because it increased antioxidant activity and was not significantly different from the 55 and 65-minute treatments. The increased antioxidant activity is caused by the steam blanching treatment, which can recover polyphenolic compounds in cocoa beans as in the water blanching treatment [36, 37]. The increase in total phenolic content due to the steam blanching treatment was most likely caused by the presence of polyphenolic compounds which did not undergo enzymatic oxidation.

Proximate parameters are significant in food and other agricultural products, including moisture, ash, fat, protein, and carbohydrates. One of the essential proximate parameters of cocoa is moisture content, which directly affects the quality and shelf life of cocoa products. The moisture content in the control was 1.15%, which gradually increased when the steaming time was also increased (Table 3). The high moisture content is caused by stretching the cell plasma due to heating during steaming. Differences in moisture content are closely related to differences in water absorption during steam blanching. Chen et al. [38] reported that during blanching, tissue softening occurs due to water absorption, which causes an increase in mass. Heras-Ramírez et al. [39] also reported that heating time could increase the diffusion of water to be more intense in making rice porridge, thus increasing the water content. The length of heating time represents the ease of migration of water molecules under certain conditions. The shorter the time, the more difficult it is for water molecules to migrate. Therefore, it is not recommended to steam cocoa beans until the interval of 65 minutes because it will further increase the moisture content of the product. High water content can accelerate product deterioration because it stimulates mould growth and affects the product's taste [40]. In this study, steaming cocoa beans is recommended at 45-minute intervals because, at that interval, the increase in moisture content is not too high, namely, only 1.45-fold compared to without steaming. Besides that, the selection at this interval is also related to the fatty acid content of snack bar products in Table 1, where the steaming time exceeds 45 minutes; the fatty acid content tends to decrease, whereas in this snack bar product, it is expected that the fatty acid content is high.

In addition, the steaming time of cocoa beans significantly reduced the ash content of snack bar products. Cocoa beans without steaming obtained an ash content of 3.15%. This amount exceeds the food safety standards for cocoa products, where the ash content allowed is ≤2.5% [32]. After steaming, the ash content was only around 1.47–2.04%. This number was lower than the ash content reported by Ilori et al. [41], which was 2.24–2.65%. The decrease in the percentage of ash was caused by the heat generated in the steaming process so that the minerals in the feedstock dissolved in water [42]. Therefore, steaming treatment is recommended to decrease the ash content of snack bar products.

Then, the steaming time significantly reduced the carbohydrate content of snack bar products, especially after the cocoa beans were steamed for 35 minutes. The decrease in carbohydrate content during steaming is caused by the main component of carbohydrates in the form of starch granules, which undergo hydrolysis due to heating during the steaming process; therefore, the carbohydrate content decreases. A decrease in the carbohydrate content in cocoa beans through heating was from 110 to 150°C by 11.76% [23]. In this study, the highest decrease occurred in the steaming time of 65 minutes, which was 17.71%. Therefore, increasing the steaming time beyond 65 minutes is not recommended.

Steaming treatment caused decreased protein contents, effectively deactivating enzymes because proteins are more easily denatured in a hot and humid environment [18]. In this study, the highest protein decrease occurred in the steaming time of 65 minutes, which was 15.3%. Therefore, increasing the steaming time beyond 65 minutes is not recommended. In this study, it is suggested that the steaming time of cocoa beans is only at 45-minute intervals because there is an increase in fatty acids, which is highly expected in snack bar products. In this study, the fat content obtained was much higher than that of cocoa in northeastern Peru, as reported by Oliva-Cruz et al. [4], which was 17.51–22.13%. The difference in fat content can be caused by geographical influences and the analytical method used [4]. Superheated steam has been used to inactivate enzymes and dry cereals such as oats, brown rice, and oats [43].

## 4. Conclusion

This study confirmed that a longer steaming time affects the fatty acids, saturated fatty acids, antioxidants, and proximate in cocoa beans. Steaming time treatment at 45 minutes increased oleic acid and palmitic acid, which are 7% and 14% higher than those without steaming treatment. In addition, reducing free fatty acids represents a quality improvement that meets international Codex Alimentarius standards, offering a competitive advantage in the market. The food industry can adopt this steaming technique to develop snack bars and new products that are healthier and more sustainable by utilizing steaming as an effective processing method in maintaining and increasing the nutritional value of products, in line with the trend of consumption and environmentally friendly. However, studies on steaming cocoa beans show the need for further research to adapt the method based on cocoa varieties and cultivation conditions, evaluate applications on an industrial scale, the impact of consumption of snack bars from steaming cacao beans on health, and consumer acceptance of the product's sensory aspects.

## Figures and Tables

**Table 1:** Fatty acids of snack bars in various steaming times of cocoa beans.

Steaming time (minutes)	Fatty acid (%)					
	Oleic (C18 : 1)	Palmitic (C16 : 1)	Stearic (C18 : 0)	Linoleic (18 : 2)	Linolenic (C18 : 3)	Myristic (C14 : 1)
0	59.48 ± 3.02b	12.76 ± 1.99d	0.09 ± 0.02e	0.95 ± 0.04c	0.046 ± 0.002e	0.003 ± 0.001e
15	62.14 ± 3.14ab	13.11 ± 1.24d	1.00 ± 0.09d	1.00 ± 0.02c	0.052 ± 0.003d	0.010 ± 0.007e
25	64.85 ± 1.72a	22.24 ± 1.17c	1.51 ± 0.07bc	1.28 ± 0.03b	0.116 ± 0.003b	0.024 ± 0.003d
35	65.35 ± 1.98a	24.15 ± 0.48b	1.64 ± 0.07b	1.39 ± 0.08ab	0.122 ± 0.002a	0.028 ± 0.004cd
45	66.79 ± 2.02a	26.69 ± 0.95a	1.83 ± 0.06a	1.52 ± 0.07a	0.125 ± 0.003a	0.035 ± 0.004bc
55	65.60 ± 2.08a	25.43 ± 0.87ab	1.62 ± 0.05b	1.43 ± 0.04ab	0.120 ± 0.004ab	0.042 ± 0.005b
65	64.96 ± 1.88a	24.44 ± 0.65b	1.47 ± 0.03c	1.28 ± 0.03b	0.095 ± 0.005c	0.054 ± 0.002a
CV (%)	3.72	4.79	6.04	9.53	2.86	16.30

*Notes*. Values are means ± standard deviation; means followed by the different letters in the same column indicate significance by Duncan multiple range test (*P* < 0.05). CV = coefficient of variance.

**Table 2:** Analysis of free fatty acids and DPPH radical-scavenging activity of snack bar products at several steaming time treatments.

Steaming time (minutes)	Variable of analysis	
	Free fatty acid (%)	DPPH IC_50_ (ppm)
0	1.090 ± 0.108a	43.99 ± 4.23a
15	0.064 ± 0.008b	35.51 ± 1.99b
25	0.060 ± 0.007b	30.47 ± 1.56c
35	0.040 ± 0.006c	29.26 ± 1.87c
45	0.020 ± 0.009d	13.81 ± 3.23d
55	0.018 ± 0.004d	13.02 ± 4.45d
65	0.015 ± 0.006d	12.72 ± 3.77d
CV (%)	6.19	10.06

*Notes*. Values are means ± standard deviation; means followed by the different letters in the same column indicate significance by Duncan multiple range test (*P* < 0.05). CV = coefficient of variance.

**Table 3:** Proximate of snack bar products.

Steaming time (minutes)	Proximate (%)				
	Moisture	Ash	Carbohydrate	Protein	Fat
0	1.15 ± 0.09c	3.15 ± 0.47a	42.52 ± 1.67a	12.61 ± 0.09a	40.09 ± 10.11a
15	1.61 ± 0.29bc	2.04 ± 0.39b	41.74 ± 0.65ab	11.69 ± 0.61b	43.87 ± 6.87a
25	1.57 ± 0.32bc	1.89 ± 0.22b	40.66 ± 0.46ab	11.43 ± 0.37bc	44.87 ± 3.84a
35	1.47 ± 0.23bc	1.88 ± 0.21b	39.21 ± 1.54bc	11.31 ± 0.32bc	46.10 ± 4.23a
45	1.67 ± 0.31b	1.80 ± 0.17b	37.14 ± 1.82cd	11.18 ± 1.23bcd	47.87 ± 3.77a
55	1.89 ± 0.43b	1.84 ± 0.15b	36.94 ± 1.97cd	11.06 ± 0.98cd	48.35 ± 4.67a
65	2.52 ± 0.21a	1.47 ± 0.11c	34.99 ± 2.01d	10.68 ± 0.91d	50.90 ± 4.67a
CV (%)	14.64	7.90	3.70	2.72	19.52

*Notes*. Values are means ± standard deviation; means followed by the different letters in the same column indicate significance by Duncan multiple range test (*P* < 0.05). CV = coefficient of variance.

## Data Availability

The data are available within the article.
